# Portable and Label-Free Quantitative Loop-Mediated Isothermal Amplification (LF-qLamp) for Reliable COVID-19 Diagnostics in Three Minutes of Reaction Time: Arduino-Based Detection System Assisted by a pH Microelectrode

**DOI:** 10.3390/bios11100386

**Published:** 2021-10-13

**Authors:** Mario Moisés Alvarez, Sergio Bravo-González, Everardo González-González, Grissel Trujillo-de Santiago

**Affiliations:** 1 Centro de Biotecnología-FEMSA, Tecnologico de Monterrey, Monterrey 64849, Mexico; A01191795@itesm.mx (S.B.-G.); dnarnaprot@gmail.com (E.G.-G.); 2 Departamento de Bioingeniería, Tecnologico de Monterrey, Monterrey 64849, Mexico; 3 Departamento de Ingeniería Mecatrónica y Eléctrica, Tecnologico de Monterrey, Monterrey 64849, Mexico

**Keywords:** SARS-CoV-2, COVID-19, qLAMP, amplification, diagnostic, microelectrodes, point-of-care

## Abstract

Loop-mediated isothermal amplification (LAMP) has been recently studied as an alternative method for cost-effective diagnostics in the context of the current COVID-19 pandemic. Recent reports document that LAMP-based diagnostic methods have a comparable sensitivity and specificity to that of RT-qPCR. We report the use of a portable Arduino-based LAMP-based amplification system assisted by pH microelectrodes for the accurate and reliable diagnosis of SARS-CoV-2 during the first 3 min of the amplification reaction. We show that this simple system enables a straightforward discrimination between samples containing or not containing artificial SARS-CoV-2 genetic material in the range of 10 to 10,000 copies per 50 µL of reaction mix. We also spiked saliva samples with SARS-CoV-2 synthetic material and corroborated that the LAMP reaction can be successfully monitored in real time using microelectrodes in saliva samples as well. These results may have profound implications for the design of real-time and portable quantitative systems for the reliable detection of viral pathogens including SARS-CoV-2.

## 1. Introduction

At present, nearly 233 million people have been diagnosed with COVID-19 worldwide, and the disease has killed more than 4.7 million people [1], making it the most lethal infectious disease in a century. COVID-19 has laid bare the severe limitations in our capacities to respond to epidemic emergencies and has made it clear that we must expand and strengthen the portfolio of available tools for effective diagnostics of infectious diseases [2] both now and in the future.

The retro-transcriptase quantitative polymerase chain reaction (RT-qPCR) is currently the gold standard methodology for COVID-19 detection [2,3,4]. Despite its unquestionable accuracy and robustness, qPCR-based methods have some serious limitations (e.g., lack of portability, dependence on centralized facilities, need for technical expertise to conduct RT-qPCR testing, and high infrastructural and operational costs) that prevent the provision of cost-effective and massive diagnostics during this time of COVID-19 [2,5,6].

One potential alternative to RT-qPCR could be loop-mediated isothermal amplification (LAMP for short), which has been extensively studied in recent reports as a cost-effective diagnostic system in the context of the current COVID-19 pandemic [7,8,9,10,11,12,13,14,15]. Overall, these reports have documented that LAMP-based methods have comparable sensitivity and specificity to that of RT-qPCR. More importantly, LAMP methods provide attractive advantages over RT-qPCR methods in terms of lower capital and operating costs. LAMP is an isothermal method, so it frees the user from the need for a costly thermal cycler. In addition, LAMP methods that exhibit excellent sensibility and acceptable specificity have been described for the extraction-free analysis of nasopharyngeal and even saliva samples [14,16,17]. The release from the need for an extraction stage further reduces the cost of LAMP methodology.

Most LAMP-based methods rely on a discrimination between positive and negative samples due to a change in the pH associated with an acidification of the reaction mix due to the liberation of hydrogen ions (H_3_O^+^) inherent in the amplification [10,14,16,18,19]. In a weakly buffered reaction medium, the change in pH may translate into a change in color if a pH indicator is added to the reaction mix. A frequent embodiment of colorimetric LAMP uses phenol red as the pH indicator [10,16,20].

However, colorimetric LAMP methods also have some limitations. One is that the adequate performance of colorimetric LAMP mediated by phenol red depends on the initial pH of the sample. This is particularly relevant in extraction-free applications, since acidic samples may prematurely shift the color of the reaction mix, even in noninfected samples, thereby possibly rendering false positives. Indeed, the direct implementation of colorimetric LAMP in saliva samples is technically challenging. Human saliva exhibits a wide range of pH values [21,22]; therefore, even small volumes of saliva may significantly affect the initial pH of the reaction mix in LAMP-based methods. Acidic saliva samples may induce a premature shift in the pH (and the color) that is not associated with amplification and will eventually lead to a false positive diagnosis. The direct and real-time reading of electric potential throughout the amplification offers a solution to some of the limitations of final-point colorimetric LAMP methods in the context of the analysis of saliva samples. LAMP-based methods also use from four to six primer sets, and the design of LAMP primer sets is nontrivial as the primers may interact among themselves during amplification. This can promote nonspecific amplifications that may also result in false positives [23,24].

In the present study, we demonstrate the straightforward use of microelectrodes to monitor, in real time, the evolution of the amplification of SARS-CoV-2 genetic material. This simple implementation renders a quantitative and label-free embodiment of the LAMP reaction (LF-LAMP) for amplification of any genetic sequence.

We show that the progressions of the signal from truly positive samples (samples containing genetic material from SARS-CoV-2) and negative samples clearly differ in LF-qLAMP. Therefore, this method provides a straightforward way to distinguish pH shifts that are truly due to specific amplifications and are not false positives.

In this time of COVID-19, we also illustrate LF-qLAMP for the diagnostics of COVID-19 from saliva samples spiked with SARS-CoV-2 nucleic acids. Overall, we demonstrate the utility of extraction-free LF-LAMP for COVID-19 from samples containing artificial SARS-CoV-2 genetic material and from saliva samples containing artificial SARS-CoV-2 genetic material.

## 2. Materials and Methods

**Figure 1:**
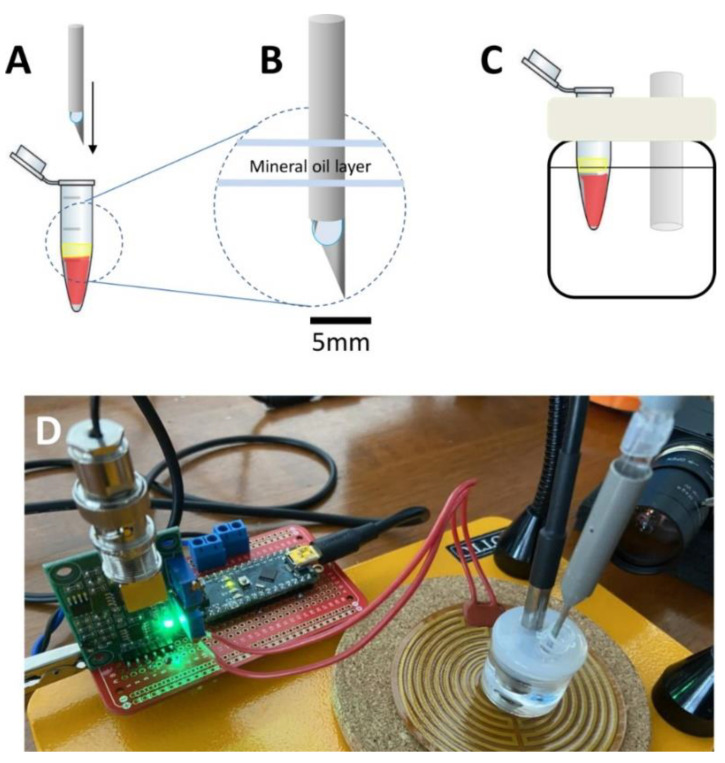
Experimental setup. (**A**) A micro pH electrode is inserted into the loop-mediated isothermal amplification (LAMP) colorimetric reaction mix and (**B**) a thin layer of oil is added to avoid evaporation during incubation (**C**) in an oil bath. (**D**) Image of the actual system; a heating mat connected to an Arduino-based proportional–integral–derivative (PID) controller is used to control the temperature at 62.5 ± 1.5 °C.

**Figure 2:**
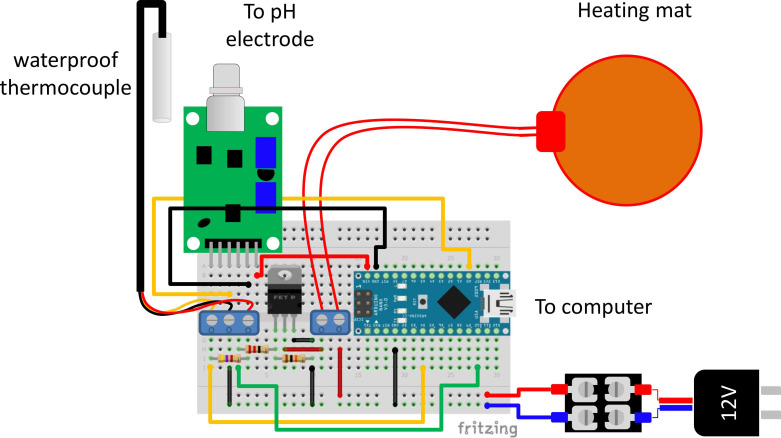
Schematic representation of the Arduino-based proportional integral derivative (PID) temperature controller and online electric potential monitoring system.

Equipment Specifications: We fabricated a simple and portable prototype for the isothermal incubation of samples and online monitoring of the LAMP amplification process using a commercial and low-cost pH sensor (GAOHOU pH 0–14; available at Amazon.com; USA; accessed on 10 October 2021), a commercial pH electrode (MI-4144B combination pH needle electrode; Microelectrodes, Inc., Bedford, NH, USA), an Arduino UNO^®^ or Arduino Nano^®^ microprocessor (Arduino, Italy), a 12 V eliminator, a 10 mL vessel with a silicon lead, and a heating mat (electric resistance) (Figure 1). The Arduino-based PID controller and monitoring unit is depicted in Figure 2 and Figure S1 and fully described in the Supplementary Material. The code used in our heating/amplification experiments has been made available as Supplementary Material (Supplementary File S1).

Experiments with synthetic genetic material: We ran several series of LF-qLAMP experiments in which samples containing different quantities of SARS-CoV-2 synthetic genetic material were analyzed. These samples were prepared by adding plasmids containing the complete N gene from SARS-CoV-2 (Integrated DNA Technologies, Coralville, IA, USA). Samples containing different concentrations of synthetic nucleic acids of SARS-CoV-2 (from 1 × 10^5^ copies to 10 copies) were prepared by successive dilutions from stocks with a concentration of 200,000 copies of the N gene of SARS-CoV-2 per µL.

These amplification experiments were conducted using the homemade system previously described for isothermal heating of the samples at 62.5 ± 1.5 °C and for online monitoring of the progression of the electric potential in the reaction mix during amplification. All reactions were conducted in a final reaction volume of 50 μL in standard 200 µL PCR-Eppendorf. Each tube contained 25.0 μL of commercial ready-mix (WarmStart^®^ Colorimetric LAMP 2× Master Mix (DNA & RNA) from New England Biolabs (Boston, MA, USA), and 14.2 µL of nuclease-free water to complete a final reaction volume of 39.2 μL. The commercial ready-mix contains phenol red as a pH indicator to reveal the shift in pH during LAMP amplification across the threshold of pH = 6.8. Independently, we prepared 8.8 µL of proprietary primer solution (3.2 μM FIP primer, 3.2 μM BIP primer, 0.4 μM F3 primer, 0.4 μM B3 primer, 0.8 μM LF primer, 0.8 μM LB primer). In each amplification experiment, 2 µL of samples were first added to an Eppendorf tube containing a reaction mix diluted with nuclease-free water. Then, 8.8 µL of the primer mix were added. Finally, the tip of the microelectrode was introduced to this reaction mix and a drop of mineral oil was added to form a surface layer and avoid evaporation.

Cleaning of the electrode tip to remove DNA: After each amplification reaction, the tip of the microelectrode was first rinsed abundantly with PBS and then immersed in a 25% Clorox^®^ solution [25,26] (1 part commercial Clorox^®^: 3 parts PBS) at 65 °C for 15 min to destroy remaining DNA that might serve as a template in the next reaction. After this treatment with Clorox, the microelectrode tip was rinsed abundantly with PBS.

Preparation of saliva samples spiked with RNA extracts: A set of 5 saliva samples obtained from healthy volunteers was spiked with different quantities of SARS-CoV-2 synthetic genetic material. Saliva samples were inactivated by heat treatment at 95.0 °C for 5 min [10,27]. Samples were collected from five volunteers after obtaining written consent. Every precaution was taken to protect the privacy of sample donors and the confidentiality of their personal information. The experimental protocol was approved on 20 May 2020 by a named institutional committee (Alfa Medical Center, Research Committee; resolution AMCCI-TECCOVID-001).

Primers used: A set of six LAMP primers, referred to here as the α-set and previously used for colorimetric LAMP [10], was designed in-house using the LAMP primer design software Primer Explorer V5 (http://primerexplorer.jp/lampv5e/index.html; accesed on 5 March 2020). These LAMP primers were designed to target a region of the sequence of the SARS-CoV-2 N gene; specifically, they were based on the analysis of alignments of the SARS-CoV-2 N gene sequences using the Geneious software (Auckland, New Zealand) (Table 1).

Analysis of the amplification trajectories: We define IP_3_, the integral of the potential with respect to time for the first 3 min of reaction (as counted from the moment of addition of primers to the reaction mix), as an indicator of the specific amplification of genetic material in the LAMP reactions Equation (1).

IP_3_ = ∫**ΔP** dt│_3 min_ (1)

**ΔP** = (ΔP_i_ − ΔP_o_) (2)

Here, ΔP_i_ is the value of electric potential measured at every sampling point and ΔP_o_ is the value of electric potential at the beginning of the experiment. Therefore, **ΔP** is the increment in potential measured at each sampling event (i.e., in our case, every second) with respect to the initial value of electric potential in the reaction mix at the initial point of the amplification. Equation (3) provides a step-by-step approximation of IP_3_, where n is the number of sampling points within the time frame from 0 to 3 min.

IP_3_ ~ Σ_i to n_ (ΔP*)_i_ (**Δt**) (3)

We also calculated the slope (m_ip3_) of the plot of IP_3_ versus time as an additional indicator of the rate of amplification.

qPCR experiments: We conducted a set of RT-qPCR to establish a correlation between CT values obtained by qPCR and IP_3_ values obtained by LF-qLAMP in samples that contained SARS-CoV-2 synthetic genetic material. The qPCR determinations were also used to validate the cleaning procedure employed to destroy and remove DNA from the microelectrode tip used in the LF-qLAMP amplifications. The quantitative amplification was conducted in a quantitative PCR thermal cycle (Rotor gene Q 5plex; Germantown, MD, USA). We amplified RNA segments of SARS-CoV-2 using two independent sets of primers (commonly referred to as N1 and N2) directed to sequences that encode the N protein of SARS-CoV-2. These primer sets have been recommended and extensively used for the diagnostics of COVID-19 in human samples [28]. The amplification mix (final volume of 20 µL) consisted of 10 µL 2× QuantiNova SYBR Green RT-Master Mix (QIAGEN, Germantown, MD, USA); 0.2 µL QN SYBR Green RT-Mix (QIAGEN, Germantown, MD, USA); 1 µL 10× primer mix (0.5 µM final concentration); and 2.0 µL of a sample containing 0, 10, 100, 1000, or 100,000 synthetic copies of the N gene of SARS-CoV-2. The amplification cycle consisted of amplification activation at 95 °C for 2 min, followed by 40 iterative cycles of denaturation for 5 s at 95 °C and combined annealing and extension for 10 s at 60 °C.

## 3. Results

### 3.1. Rationale of the Design and Operation

Label-free quantitative LAMP (LF-qLAMP) allows the continuous and online monitoring of the change in the electric potential of the reaction mix due to the release of hydrogen ions during amplification. The release of H_3_O^+^ ions inherent in the amplification process can be measured in real time as a difference in the electric potential [29,30] or a pH change [31,32].

We fabricated a simple and portable prototype to accomplish two purposes: the isothermal incubation of samples and the online monitoring of the amplification process. We used a simple incubator, a PID temperature controller, a commercial and low-cost pH sensor, and a commercial pH microelectrode. Both the PID-temperature controller and the online pH sensing system were enabled by an Arduino UNO^®^ or Arduino Nano^®^ microprocessor.

Figure 1 and Figure 2 show the different aspects of this prototype. The isothermal amplification reactions were conducted at 62.5 °C ± 1.5 °C in 200 µL Eppendorf tubes immersed in a mineral oil bath (Figure 1A) and containing a total reaction mix volume of 50 µL. The tip of a commercial pH microelectrode was immersed into the reaction mix for online monitoring of the progression of the electric potential (in mV) using an Arduino-based system. A simple Arduino code was used to enable the collection and transmission of data to the computer (Supplementary File S1); the difference in the electric potential (ΔP), as evaluated in the reaction solution, was continuously transmitted and recorded at a sampling rate of 1 Hz (i.e., 1 sampling point per second). In our experiments, the insertion of the electrode within the reaction mix impedes closure of the Eppendorf tube during incubation. To avoid evaporation during heating, two drops of mineral oil were dispensed into each Eppendorf tube before electrode placement to allow the development of a thin lid layer of mineral oil at the liquid surface of the reaction mix. The stability of the electric potential readings is affected by temperature fluctuations around the set point. Therefore, adequate temperature control (62.5 ± 1.5 °C) is highly recommended for conclusive results. In our experiments, mostly executed at home, the temperature within the oil bath was controlled at 62.5 °C using an Arduino-based PID controller. Alternatively, the LAMP reaction can be incubated in any commercial thermoblock or in a miniPCR^®^ apparatus [33].

Figure 2 schematically shows the connections that should be made between the Arduino microcontroller, the low-cost pH sensor, and the rest of the components of the circuit that constitute the PID control system and the online electric potential monitor. Figure S1 shows an image of the actual experimental Arduino-based PID controller system.

### 3.2. Characterization of the Performance of the Monitoring System

**Figure 3:**
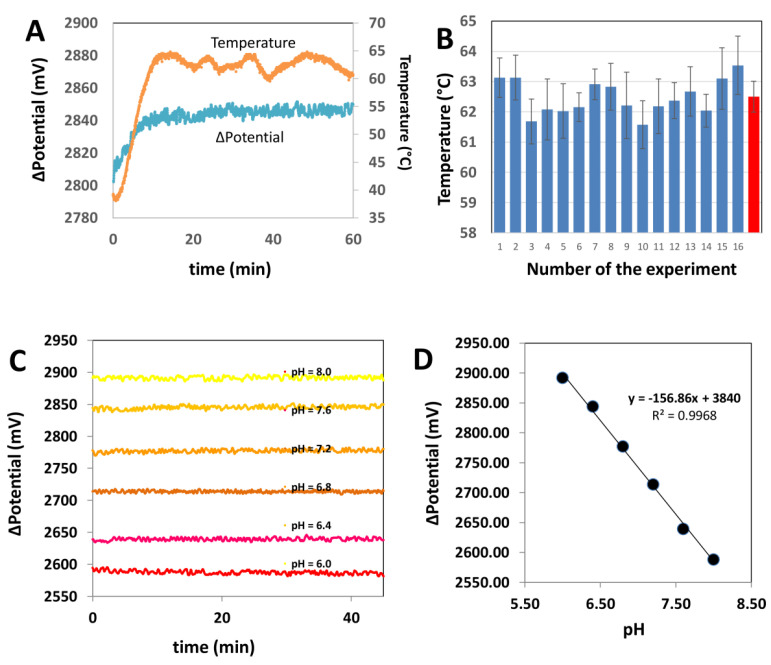
Basic characterization of the electric potential monitoring system and the Arduino-based proportional–integral–derivative (PID) controller. (**A**) The potential differential is stable at isothermal conditions. The evolution of the potential differential is marked in blue. Temperature is marked in orange. (**B**) The Arduino-based PID controller is able to maintain the temperature value around the set point value (62.5 ± 0.5 °C) in a set of 16 independent experiments indicated with blue bars. The red bar indicates the average temperature and standard deviations of this set of 16 independent experiments. (**C**) The potential signal is stable at different pH values in the range of 6.0 to 8.0. (**D**) A linear dependence between pH and potential differential was observed in the entire range of pH values relevant to the Loop-mediated Isothermal Amplification (LAMP) reaction.

We evaluated the stability of the potential signal at the temperature set point and during temperature ramps. The electric potential is a strong function of the temperature (Figure 3A). For instance, the electric potential increased ~90 mV per 10 °C in our experiments in the range of 45 to 55 °C. By contrast, at isothermal conditions, the intrinsic variation of the electric potential was very stable; the variations were lower than 0.3% for the range of pH buffers that we assayed.

We also analyzed the ability of this simple PID controller to function for extended incubation periods (i.e., 60 min) and under different room temperatures (Figure 3B). Overall, we found that this simple incubator system is sufficiently robust to maintain the temperature at the set point (average temperature of 62.5 °C; average standard deviation of 0.5 °C) regardless of the room temperature (i.e., in a range between 15 and 25 °C). As we will show, this level of control is sufficient to obtain reliable and reproducible results for determination of the electric potential in solutions with different pH values and during LAMP reactions.

In a first set of experiments, we evaluated the robustness and consistency of the characteristics of the electrode readings at different pH values. Figure 3C,D shows representative readings associated with the immersion of the microelectrode in buffer solution of different pH values (i.e., pH 6.0, 6.4, 6.8, 7.2, 7.6, and 8.0).

The standard deviation associated with the readings within this window of pH values ranges from 1.38 and 6.91 mV (Table S1). For example, the average electric potential measured for a buffer solution at pH 6.8 (which is approximately the threshold value of phenol red) was 2777 mV and exhibited a standard deviation of ±1.38 mV. A buffer solution at pH 8.0 (which is similar to the pH value of the LAMP buffer used) had an electric potential of 2587.58 mV with a standard deviation of ±6.21 mV. Overall, we observed coefficients of variance lower than 0.3% for all the experiments conducted in the absence of amplification reactions. In all these cases, the electric potential remains essentially stable during incubation periods of 60 min. This indicates that the inherent error (i.e., the intrinsic fluctuation associated with the potential signal at the experimental conditions) is in the range of ±12.3 mV (i.e., the maximum standard deviations are lower than 7 mV).

### 3.3. LF-qLAMP in Saliva Samples: Discrimination between Positive and Negative Samples

As already mentioned, colorimetric LAMP using phenol red has recently received attention as a cost-effective diagnostic method that enables discrimination between SARS-CoV-2 positive and SARS-CoV-2 negative samples by naked eye inspection and is therefore independent of the use of costly PCR instruments [10,34,35].

However, the direct implementation of colorimetric LAMP in saliva samples is technically challenging. Human saliva exhibits a wide range of pH values [21,22]; therefore, even small volumes of saliva may significantly affect the initial pH of the reaction mix in LAMP-based methods. Acidic saliva samples may induce a premature shift in the pH (and the color) that is not associated with amplification and will eventually lead to a false positive diagnosis. The direct and real-time reading of electric potential throughout the amplification offers a solution to some of the limitations of final-point colorimetric LAMP methods in the context of the analysis of saliva samples.

Using the simple Arduino-based system described, we incubated a series of LAMP reactions and monitored the progress of the amplification reaction by measuring the electric potential in the reaction mix in real time. We successfully discriminated between SARS-CoV-2 positive samples (i.e., those that contain SARS-CoV-2 genetic material) and SARS-CoV-2 negative samples (i.e., those that do not contain SARS-CoV-2 genetic material) in a set of saliva samples spiked with synthetic SARS-CoV-2 DNA.

We first characterized the baseline corresponding to negative samples, where no primers were added (Figure S2), As expected, the signal associated with these samples was relatively steady (average standard deviations ~10.84; *n* = 4). We then monitored the evolution of the LAMP reactions in the negative samples containing LAMP reaction mix and primers, but no synthetic SARS-CoV-2 genetic material.

One of the limitations of colorimetric LAMP-based methods based on the phenol red color shift is that some degree of color change exists in samples containing no template (see ref [29]). This has been associated to the occurrence of nonspecific amplification induced by the interaction among the LAMP primers [24,34], which often serve as templates for off-target polymerizations. In general, nonspecific amplification is believed to be the main reason for false positives in LAMP colorimetric reactions.

**Figure 4:**
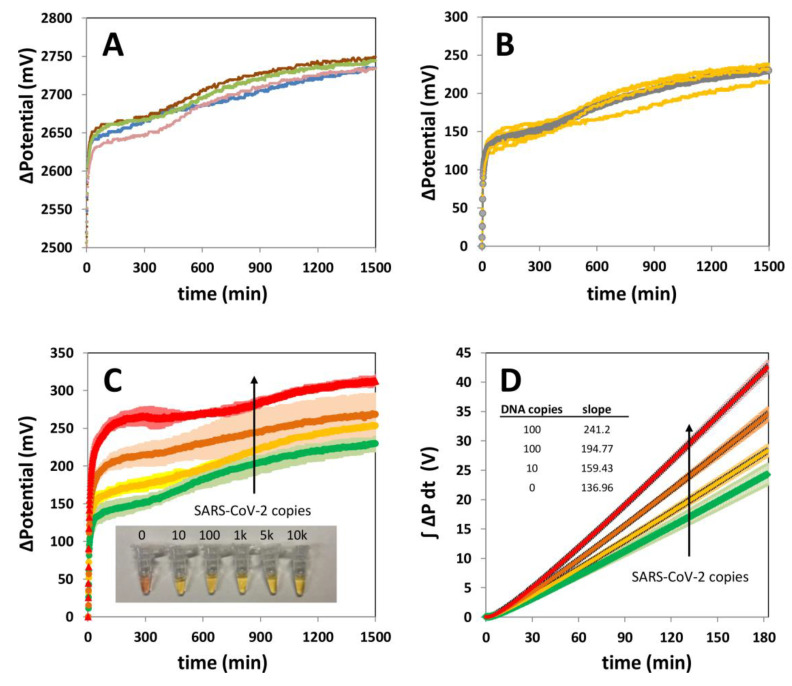
Discrimination between SARS-CoV-2 positive and negative saliva samples using online electric potential measurements. (**A**) Measurement of four different negative samples (i.e., samples without SARS-CoV-2 but containing reaction mix and primers). (**B**) Normalized signal of a set of four different negative saliva samples indicated with yellow lines. Values were normalized by subtracting the initial potential value of each curve. The grey line indicates the average of the four yellow trajectories (yellow lines) and is characteristic of the progression of the Loop-mediated Isothermal Amplification (LAMP) reaction observed in negative samples. (**C**) Comparison of the online potential signal in saliva samples containing 1000 (red), 100 (orange), and 10 (yellow) copies of synthetic SARS-CoV-2 genetic material. The average of four negative samples is depicted in green. The inset shows a close-up of the typical end point color of a negative sample and several positive samples (which contained different quantities of SARS-CoV-2 genetic material) during the amplification reaction. Shaded areas indicate standard deviations, as calculated pointwise for four independent replicates. (**D**) Plot of the average values of IP_3_ (the integral of the difference of potential with respect to time for the first three minutes of amplification) in samples containing different copy numbers of synthetic SARS-CoV-2 genetic material: 1000 (red; *n* = 4), 100 (orange; *n* = 4), and 10 (yellow; *n* = 6) copies of synthetic SARS-CoV-2 DNA. The typical trend associated with negative samples is presented in green (*n* = 6). The standard deviations, as calculated pointwise for four independent replicates, are indicated with error bars. The values of the slope associated with each trend are presented within the inset.

Figure 4A shows the typical profile of the progression of the electric potential in negative samples (with primers and reaction mix but no SARS-CoV-2 template). Four representative replicates are shown. The shape of the progression of the electric potential in the negative samples is highly consistent. Figure 4B presents a normalization of the four representative curves shown in Figure 4A. The curves were normalized by subtracting the initial potential value for each curve. In doing so, all curves practically collapse into an invariant shape. The grey line in Figure 4B depicts the average of the four curves corresponding to independently run negative samples. This profile, characteristic of negative samples, is distinguishable from that inherent to the specific amplification observed in samples that do contain SARS-CoV-2 genetic material.

Figure 4C shows the trajectories of the electric potential associated with positive samples containing different quantities of SARS-CoV-2 synthetic genetic material (color curves; i.e., in the range of 10 to 1000 copies of the N gene of SARS-CoV-2).

In this experimental set, the reaction mix contained 2–3 µL of real undiluted saliva (volume defined based in ref [14]) from SARS-CoV-2 negative volunteers (as determined by end-point colorimetric LAMP and or qPCR). Figure 4C shows that the electrical potential in the reaction mix exhibits a distinctive, practically immediate, and sharp increase at the incubation conditions upon the addition of primers. This increase is associated with a high initial rate of amplification. Noticeably, the initial slope of the potential curve is steeper in positive samples than in negative samples. The typical smoother progression associated with negative samples (Figure 4B) was also observed in negative saliva samples; the average progression of the electric potential in negative samples is indicated by the green line. The amplification curves that corresponded to samples spiked with SARS-CoV-2 genetic material exhibited a steep slope that was immediately observable upon initiation of the reaction by the addition of primers. A high sampling rate is required in the monitoring device for a clear identification of this steep initial slope. In our experiments, we achieved the resolution needed to identify the sharp slope associated with samples that contained SARS-CoV-2 genetic material by sampling at least once per second (the Arduino code is available in Supplementary File S1). Lower sampling rates (e.g., 0.2 Hz) do not serve the purpose of clearly resolving the initial slope of the reaction in positive samples (Figure S3). We also observed that the evolution of each curve is consistent with the copy load in the positive samples, so that higher copy numbers originate curves with a higher initial slope than is observed for curves associated with lower loads. Since the initial rate of amplification is remarkably high in positive samples (>25 to 75 mV s^−1^), the differences between slopes can be clearly established only if high sampling rates (~1 sample s^−1^: 1 Hz) are imposed.

The discrimination between negative samples and positive samples with medium to high viral loads (i.e., at least 100 copies) was feasible by comparison of the potential trajectories. However, samples containing a low copy number (i.e., less than 10 gene copies) cannot be distinguished from negative samples by only comparing trajectories (Figure 4C). The behavior of the trajectory of the amplification reaction in positive and negative samples can be better discriminated during the first stage of the amplification (i.e., the first three minutes of the amplification). Indeed, the value of the area under the curve or of ΔP versus t is distinguishably higher in positive samples than in negative samples during the first stage of the amplification.

We defined IP_3_ (Equation (1)), the integral of the potential with respect to time for the first 3 min of reaction, as an indicator of the specific amplification of genetic material in the LAMP reactions (Materials and Methods).

We found that IP_3_ is a reliable and robust predictor that enables the consistent and rapid identification of samples containing (or not containing) SARS-CoV-2 genetic material. Figure 4D shows the evolution of the integral of IP_3_ for the first 3 min of a set of LAMP reactions. Progressions corresponding to samples added with different quantities of synthetic genetic material (i.e., 10, 100, and 1000 copies of the N gene of SARS-CoV-2) were compared versus the average trend of six independent repeats of negative samples (i.e., samples without SARS-CoV-2 genetic material). Negative samples (plotted in green) exhibit integrals significantly lower than those calculated from the analysis of positive samples.

**Figure 5:**
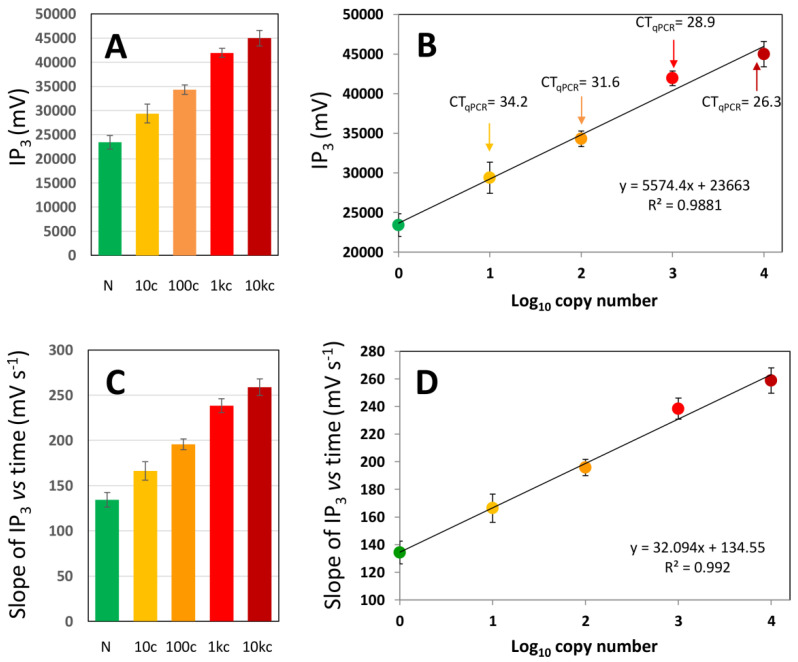
Results from the online evaluation of electric potential in five saliva samples from volunteers diagnosed as SARS-CoV-2 negative (green symbols) and a sets of five saliva samples added with 10 (yellow), 100 (orange), 1000 (red), and 10,000 copies (burgundy symbols) of SARS-CoV-2 genetic material. (**A**) Value of the integral of the differential of potential with respect to time for the first 3 min of amplification (defined here as IP_3_ value) in saliva samples containing different copy numbers of synthetic SARS-CoV-2 genetic material in the range of 10–10,000 copies (yellow, orange, red, and burgundy bars) or not containing SARS-CoV-2 genetic material (green bar). (**B**) A correlation exists between the number of synthetic N gene copies of SARS-CoV-2 in the sample and the IP_3_ value. The corresponding cycle threshold (CT) values, as determined by qPCR, are indicated with arrows. (**C**) Value of the slope of the plot of IP_3_ versus time for the same set of samples; (**D**) a correlation also exists between the slope of the IP_3_ time plot and the gene copy number in the sample. In all cases, data from at least four independent runs were analyzed. Error bars indicate standard deviations.

In Figure 5A, we have plotted the results from a set of experiments in which the IP_3_ values for saliva samples with or without SARS-CoV-2 genetic material were calculated. The IP_3_ values were clearly higher in positive samples than in negative samples. The positive samples with low to medium SARS-CoV-2 gene copy loads were also clearly distinguishable from negative samples. In addition, a linear correlation can be established between the logarithm of the copy number and the value of the integral (Figure 5B). Similarly, the plot of IP_3_ versus time is practically a straight line (Figure 4D); the value of the slope of this plot also correlates with the number of SARS-CoV-2 gene copies originally present in a sample (Figure 5C,D). These results suggest that IP_3_ (and the slope of the linear plot of IP_3_ versus time) are reliable and quantitative indicators of the specific amplification of genetic material in saliva samples.

The IP_3_ value is an indicator of the extent of specific amplification that is somehow analogous to the progression of the change of color (from red–magenta to crisp yellow; Figure S4) during the amplification in colorimetric LAMP. However, the time scale at which color and electric potential evolve differs quite drastically. Changes in color are observable at a much slower rate—positive and negative samples can be discriminated due to their color evolution within the first 30 min of reaction (Figure S4A). Remarkably, the evolution of the IP_3_ indicator introduced here enables a reliable discrimination between positive and negative samples within the initial 3 min of the reaction.

## 4. Discussion

We demonstrated the use a portable Arduino-based LAMP system as a label-free quantitative method (LF-qLAMP) for the reliable and fast identification of samples containing SARS-CoV-2 genetic material. We introduced a simple and fully portable setup for the detection of viral genetic material using commercially available pH microelectrodes for real-time monitoring of the progression of LAMP reactions. Remarkably, this LAMP-based method is capable of discerning between positive and negative samples during the first three minutes of amplification.

The result presented may be highly relevant in practical terms. The typical concentration of gene copies of SARS-CoV-2 in the saliva of positive subjects (even asymptomatic ones) is generally higher than 10^5^ mL^−1^ (10^2^ µL^−1^). In the analysis presented here, 2 µL of undiluted saliva can be added without observing inhibitory effects in terms of the extent of the amplification. Therefore, copy number values greater than 10^2^ per assay can be expected. Based on our experiments, the limit of detection/discrimination of this method, assisted by online monitoring of the electric potential of the solution during amplification, is one orders of magnitude below this (i.e., 10 SARS-CoV-2 copies per assay), suggesting that the presented method could render reliable diagnosis of COVID-19 in unextracted (undiluted or diluted) saliva samples that have even moderate or low viral loads. This hypothesis remains to be tested by the diagnostic evaluation of saliva samples from actual patients and, at this point, should be taken as an inference.

Several limitations of the methodology presented here should also be noted. The successful performance of this diagnostic strategy is centered on the ability to sample the electric potential (or pH) of the reaction mix frequently (the authors recommend at least 1.0 Hz) during the initial stage of the amplification reaction (i.e., at least during the initial three minutes). Failure to do this may hinder the resolution of the readings during the first seconds of the amplification due to a lag in the response or the adjustment of the sensor to the pH conditions (Figure S3). Further, in the current developmental state of our diagnostic device, we depend on the use of commercial pH microelectrodes that are relatively expensive. The development of disposable and low-cost pH microelectrodes should be a priority for enabling the cost-effective use of this strategy to intensify the ability to perform label-free and portable/mobile molecular diagnostics of SARS-CoV-2 and other pathogens.

## 5. Conclusions

Here, we introduced a strategy for reliable and consistent identification of samples containing SARS-CoV-2 genetic material. This strategy relies on the real-time monitoring of the LAMP reaction in the weakly buffered reaction mix. The continuous release of protons associated with the incorporation of bases into the nascent chains of DNA during amplification is measured online with a pH microelectrode that is immersed in the reaction mix incubated at 62.5 °C. We also showed that proper isothermal control and online determination of changes in potential (or pH) in the LAMP reaction mix can be accomplished using a simple Arduino-based device.

We also demonstrate that IP_3_—defined here as the area under the curve that describes the time evolution of the electric potential of the reaction mix during the first three minutes of the amplification—is a reliable predictor of the extent of specific amplification occurring during the LAMP reactions. The evaluation of IP_3_ enables the consistent discrimination between samples containing or not containing SARS-CoV-2 genetic material. In addition, we demonstrated the feasibility of using this portable, fast, and reliable diagnostic strategy with saliva samples containing different loads of SARS-CoV-2 genetic material.

Recent reports have shown that the performance of LAMP is greatly influenced by the reaction conditions and the reagents used (i.e., primers sets, retro-transcriptases, or proteases, additives, and fluorescent dyes, among others) [16,36]. The label-free qLAMP platform presented here may be highly useful in the selection of reaction reagents (i.e., alternative polymerases, additives to enhance the amplification, or primer sets) or optimization of LAMP conditions (i.e., temperatures and concentrations). The mere comparison of the curves of the evolution of protons with time under different reaction conditions provides a direct way to evaluate the effect of any modifications in the protocol on the overall performance of the amplification.

The release of protons during amplification is inherent in all nucleic acid amplification methods. Therefore, the methodology of real-time monitoring of the change in electric potential during amplification is fully translatable to other amplification schemes (i.e., qPCR and RPA, among others).

## Figures and Tables

**Table 1:** Sequences of the Loop-mediated Isothermal Amplification (LAMP) primers used for the detection of segments of genetic material encoding for the expression of the N protein of SARS-CoV-2.

Set	Description	Primers Sequence
Primer set α (LAMP)	2019-nCoV 1-F3	*TGGACCCCAAAATCAGCG*
	2019-nCoV 1-B3	*GCCTTGTCCTCGAGGGAAT*
	2019-nCoV 1-FIP	CCATGCGTTCTCCATTCTGGTAAATGCACCCCCATTACG
	2019-nCoV 1-BIP	*CGCGATCAAAACAACGTCGGCCCTTGCCATGTTGAGTGAGA*
	2019-nCoV 1-LF	*TGAATCTGAGGGTCCACCAA*
	2019-nCoV 1-LB	*TTACCCAATAATACTGCGTCTTGGT*

## Data Availability

The data presented in this study are openly available in FigShare at DOI:10.6084/m9.figshare.16779208.

## Supplementary Materials

The following are available online at https://www.mdpi.com/article/10.3390/bios11100386/s1. Figure S1: Actual image of the Arduino-based proportional integral derivative (PID) temperature controller and the online pH/electric potential monitoring system used in our experiments; Figure S2: Readings associated with negatives samples; Figure S3: Progression of the electric potential with time for negative samples and positive samples containing copies of synthetic SARS-CoV-2 genetic material collected at suboptimal sampling rate; Figure S4: Real-time determination of color changes; Table S1: Average electric potential associated with buffers of different pH values; File S1: Arduino code needed for eLAMP.

## References

[1] Home—Johns Hopkins Coronavirus Resource Center. https://coronavirus.jhu.edu/.

[2] Esbin M.N., Whitney O.N., Chong S., Maurer A., Darzacq X., Tjian R. (2020). Overcoming the bottleneck to widespread testing: A rapid review of nucleic acid testing approaches for COVID-19 detection. RNA.

[3] Younes N., Al-Sadeq D.W., AL-Jighefee H., Younes S., Al-Jamal O., Daas H.I., Yassine H.M., Nasrallah G.K. (2020). Challenges in Laboratory Diagnosis of the Novel Coronavirus SARS-CoV-2. Viruses.

[4] Cheng M.P., Papenburg J., Desjardins M., Kanjilal S., Quach C., Libman M., Dittrich S., Yansouni C.P. (2020). Diagnostic Testing for Severe Acute Respiratory Syndrome-Related Coronavirus 2: A Narrative Review. Ann. Intern. Med..

[5] Sharfstein J.M., Becker S.J., Mello M.M. (2020). Diagnostic Testing for the Novel Coronavirus. JAMA J. Am. Med. Assoc..

[6] Giri A.K., Rana D.R. (2020). Charting the challenges behind the testing of COVID-19 in developing countries: Nepal as a case study. Biosaf. Health.

[7] Yu L., Wu S., Hao X., Dong X., Mao L., Pelechano V., Chen W.-H., Yin X. (2020). Rapid Detection of COVID-19 Coronavirus Using a Reverse Transcriptional Loop-Mediated Isothermal Amplification (RT-LAMP) Diagnostic Platform. Clin. Chem..

[8] Lamb L.E., Bartolone S.N., Ward E., Chancellor M.B. (2020). Rapid Detection of Novel Coronavirus (COVID-19) by Reverse Transcription-Loop-Mediated Isothermal Amplification. SSRN Electron. J..

[9] Zhang Y., Odiwuor N., Xiong J., Sun L., Nyaruaba R.O., Wei H., Tanner N.A. (2020). Rapid Molecular Detection of SARS-CoV-2 (COVID-19) Virus RNA Using Colorimetric LAMP. medRxiv.

[10] González-González E., Lara-Mayorga I.M., Rodríguez-Sánchez I.P., Zhang Y.S., Martínez-Chapa S.O., Santiago G.T., Alvarez M.M. (2021). Colorimetric loop-mediated isothermal amplification (LAMP) for cost-effective and quantitative detection of SARS-CoV-2: The change in color in LAMP-based assays quantitatively correlates with viral copy number. Anal. Methods.

[11] Rodriguez-Manzano J., Malpartida-Cardenas K., Moser N., Pennisi I., Cavuto M., Miglietta L., Moniri A., Penn R., Satta G., Randell P. (2021). Handheld Point-of-Care System for Rapid Detection of SARS-CoV-2 Extracted RNA in under 20 min. ACS Cent. Sci..

[12] Zhu X., Wang X., Han L., Chen T., Wang L., Li H., Li S., He L., Fu X., Chen S. (2020). Multiplex reverse transcription loop-mediated isothermal amplification combined with nanoparticle-based lateral flow biosensor for the diagnosis of COVID-19. Biosens. Bioelectron..

[13] Toppings N., Mohon A., Lee Y., Kumar H., Lee D., Kapoor R., Singh G., Oberding L., Abdullah O., Kim K. (2021). Saliva-Dry LAMP: A Rapid Near-Patient Detection System for SARS-CoV-2. Res. Square.

[14] Lalli M.A., Langmade J.S., Chen X., Fronick C.C., Sawyer C.S., Burcea L.C., Wilkinson M.N., Fulton R.S., Heinz M., Buchser W.J. (2021). Rapid and Extraction-Free Detection of SARS-CoV-2 from Saliva by Colorimetric Reverse-Transcription Loop-Mediated Isothermal Amplification. Clin. Chem..

[15] Rabe B.A., Cepko C. (2020). SARS-CoV-2 detection using isothermal amplification and a rapid, inexpensive protocol for sample inactivation and purification. Proc. Natl. Acad. Sci. USA.

[16] Alekseenko A., Barrett D., Pareja-Sanchez Y., Howard R.J., Strandback E., Ampah-Korsah H., Rovšnik U., Zuniga-Veliz S., Klenov A., Malloo J. (2021). Direct detection of SARS-CoV-2 using non-commercial RT-LAMP reagents on heat-inactivated samples. Sci. Rep..

[17] L’Helgouach N., Champigneux P., Schneider F.S., Molina L., Espeut J., Alali M., Baptiste J., Cardeur L., Dubuc B., Foulongne V. (2020). EasyCOV: LAMP based rapid detection of SARS-CoV-2 in saliva. medRxiv.

[18] Zhao J., Gao J., Zheng T., Yang Z., Chai Y., Chen S., Yuan R., Xu W. (2018). Highly sensitive electrochemical assay for Nosema bombycis gene DNA PTP1 via conformational switch of DNA nanostructures regulated by H+ from LAMP. Biosens. Bioelectron..

[19] Rothberg J.M., Hinz W., Rearick T.M., Schultz J., Mileski W., Davey M., Leamon J.H., Johnson K., Milgrew M.J., Edwards M. (2011). An integrated semiconductor device enabling non-optical genome sequencing. Nature.

[20] Tanner N.A., Zhang Y., Evans T.C. (2015). Visual detection of isothermal nucleic acid amplification using pH-sensitive dyes. Biotechniques.

[21] Cohen M., Khalaila R. (2014). Saliva pH as a biomarker of exam stress and a predictor of exam performance. J. Psychosom. Res..

[22] Baliga S., Muglikar S., Kale R. (2013). Salivary pH: A diagnostic biomarker. J. Indian Soc. Periodontol..

[23] Rolando J.C., Jue E., Barlow J.T., Ismagilov R.F. (2021). Real-time kinetics and high-resolution melt curves in single-molecule digital LAMP to differentiate and study specific and non-specific amplification. Nucleic Acids Res..

[24] Gao X., Sun B., Guan Y. (2019). Pullulan reduces the non-specific amplification of loop-mediated isothermal amplification (LAMP). Anal. Bioanal. Chem..

[25] Prince A.M., Andrus L. (1992). PCR: How to kill unwanted DNA. Biotechniques.

[26] Kampmann M.L., Børsting C., Morling N. (2017). Decrease DNA contamination in the laboratories. Forensic Sci. Int. Genet. Suppl. Ser..

[27] Kobayashi G.S., Brito L.A., de Moreira D.P., Suzuki A.M., Hsia G.S.P., Pimentel L.F., de Paiva A.P.B., Dias C.R., Lourenço N.C.V., Oliveira B.A. (2021). A Novel Saliva RT-LAMP Workflow for Rapid Identification of COVID-19 Cases and Restraining Viral Spread. Diagnostics.

[28] Lu X., Wang L., Sakthivel S.K., Whitaker B., Murray J., Kamili S., Lynch B., Malapati L., Burke S.A., Harcourt J. (2020). US CDC Real-Time Reverse Transcription PCR Panel for Detection of Severe Acute Respiratory Syndrome Coronavirus 2. Emerg. Infect. Dis..

[29] Gosselin D., Gougis M., Baque M., Navarro F.P., Belgacem M.N., Chaussy D., Bourdat A.G., Mailley P., Berthier J. (2017). Screen-Printed Polyaniline-Based Electrodes for the Real-Time Monitoring of Loop-Mediated Isothermal Amplification Reactions. Anal. Chem..

[30] Han D., Chand R., Kim Y.S. (2017). Microscale loop-mediated isothermal amplification of viral DNA with real-time monitoring on solution-gated graphene FET microchip. Biosens. Bioelectron..

[31] Toumazou C., Shepherd L.M., Reed S.C., Chen G.I., Patel A., Garner D.M., Wang C.J.A., Ou C.P., Amin-Desai K., Athanasiou P. (2013). Simultaneous DNA amplification and detection using a pH-sensing semiconductor system. Nat. Methods.

[32] Xie S., Yuan Y., Song Y., Zhuo Y., Li T., Chai Y., Yuan R. (2014). Using the ubiquitous pH meter combined with a loop mediated isothermal amplification method for facile and sensitive detection of Nosema bombycis genomic DNA PTP1. Chem. Commun..

[33] González-González E., Trujillo-de Santiago G., Lara-Mayorga I.M., Martínez-Chapa S.O., Alvarez M.M. (2020). Portable and accurate diagnostics for COVID-19: Combined use of the miniPCR thermocycler and a well-plate reader for SARS-CoV-2 virus detection. PLoS ONE.

[34] Wong Y.-P., Othman S., Lau Y.-L., Radu S., Chee H.-Y. (2018). Loop-mediated isothermal amplification (LAMP): A versatile technique for detection of micro-organisms. J. Appl. Microbiol..

[35] Nawattanapaiboon K., Pasomsub E., Prombun P., Wongbunmak A., Jenjitwanich A., Mahasupachai P., Vetcho P., Chayrach C., Manatjaroenlap N., Samphaongern C. (2021). Colorimetric reverse transcription loop-mediated isothermal amplification (RT-LAMP) as a visual diagnostic platform for the detection of the emerging coronavirus SARS-CoV-2. Analyst.

[36] Janíková M., Hodosy J., Boor P., Klempa B., Celec P. (2021). Loop-mediated isothermal amplification for the detection of SARS-CoV-2 in saliva. Microb. Biotechnol..

