# Supplementary material for: Portable and Label-Free Quantitative Loop-Mediated Isothermal Amplification (LF-qLamp) for Reliable COVID-19 Diagnostics in Three Minutes of Reaction Time: Arduino-Based Detection System Assisted by a pH Microelectrode
Source: Biosensors (Basel). 2021 Oct 13;11(10):386. doi: 10.3390/bios11100386 (PMC8533988; doi:10.3390/bios11100386)
Supplement: Supplementary file 1 [file biosensors-11-00386-s001.zip › biosensors-1355504-supplementary.pdf]

biosensors

MDPI

*Supplementary Materials*

# Portable and Label-Free Quantitative Loop-Mediated Isothermal Amplification (LF-qLamp) for Reliable COVID-19 Diagnostics in Three Minutes of Reaction Time: Arduino-Based Detection System Assisted by a pH Microelectrode

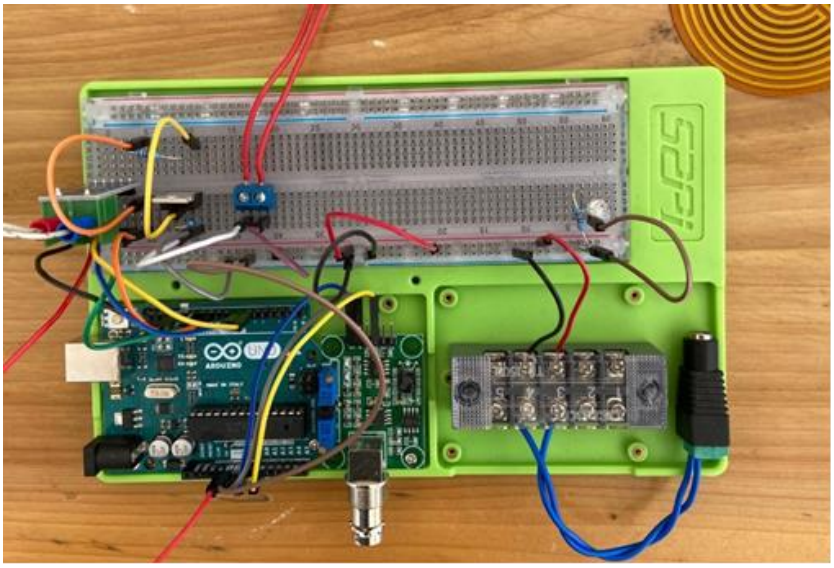

(**a**)

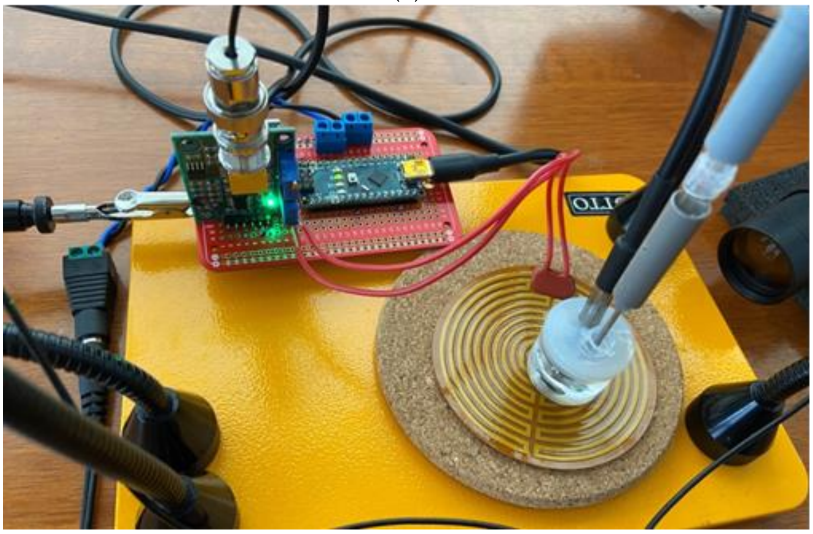

(**b**)

**Figure S1.** Actual images of the Arduino-based proportional integral derivative (PID) temperature controller and the online pH/electric potential monitoring system used in our experiments. (**A**) System based on Arduino Uno, and (**B**) system based on Arduino Nano.

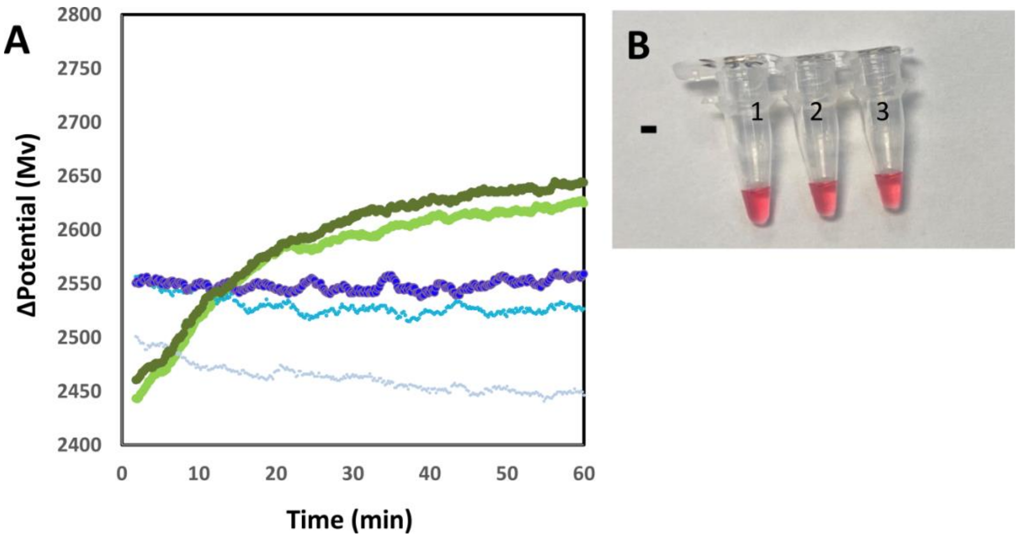


**Figure S2.** Readings associated with negatives samples. (**A**) Samples without primers exhibit a constant electrical potential (blue shaded lines); (**B**) Samples added with primers exhibit an increasing progression in the value of electrical potential as non-specific amplification occurs. Negative samples preserve their red color in colorimetric LAMP.

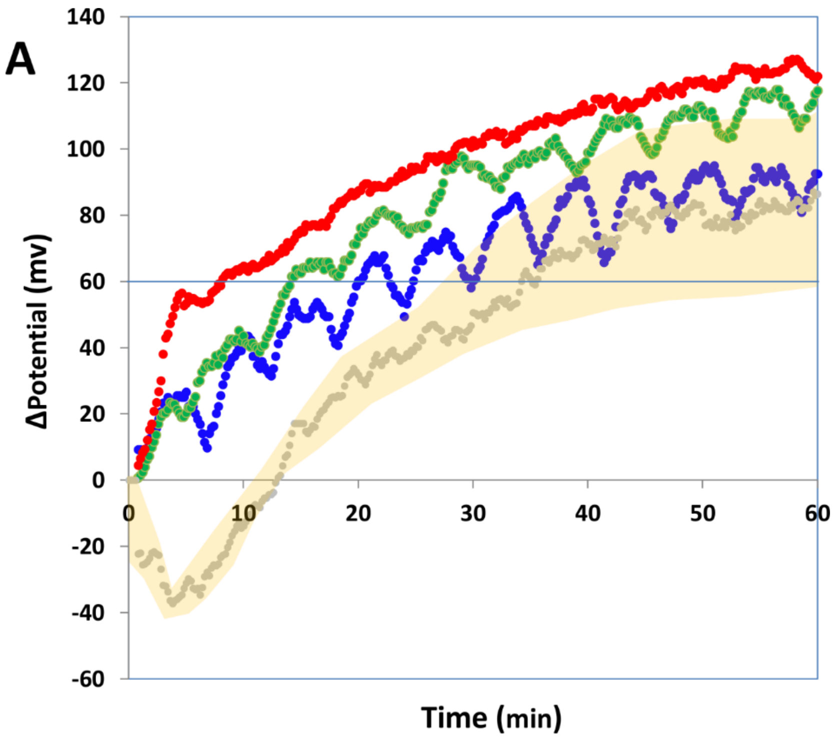


**Figure S3.** Progression of the electric potential with time for negative samples (beige trend) and positive samples containing copies of synthetic SARS-CoV-2 genetic material collected at suboptimal sampling rate (100 copies, blue trend; 1000 copies, green trend; 1000 copies, red trend). The shade in the beige trend associated to negative samples indicates standard deviation.

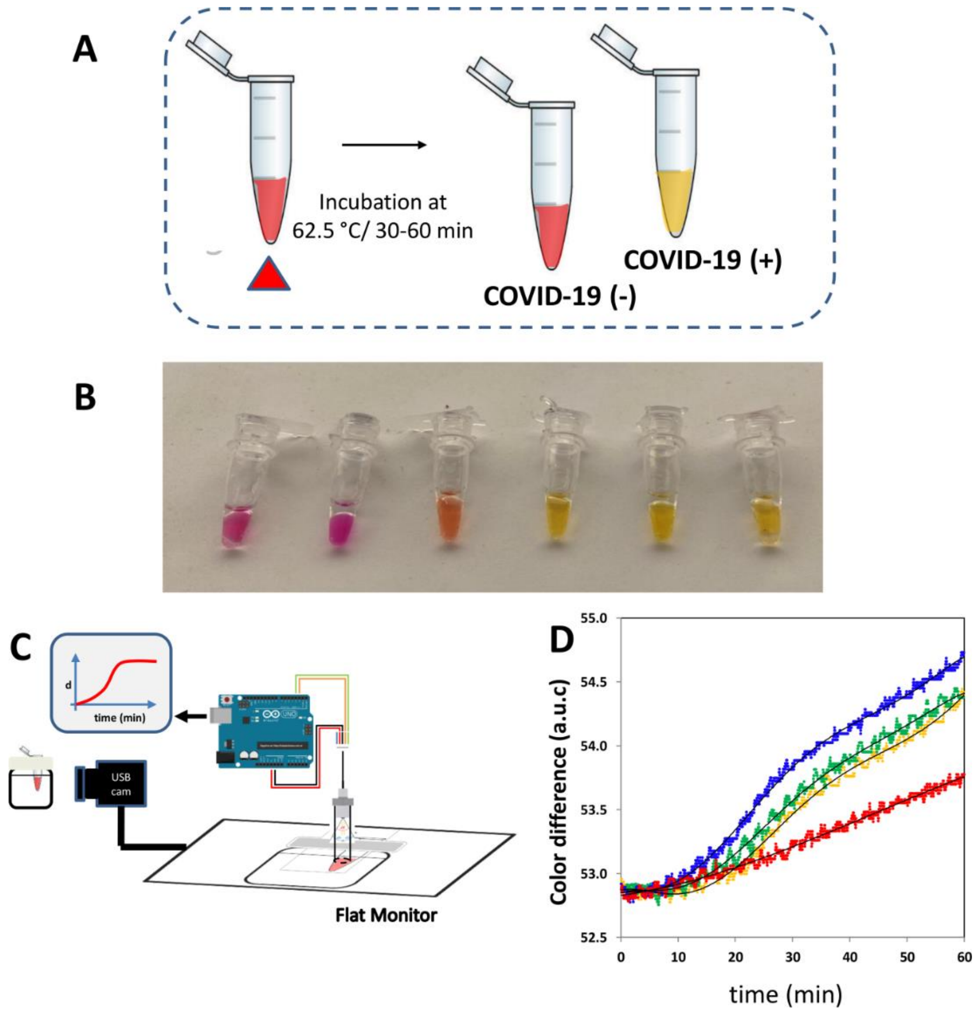


**Figure S4.** Real-time determination of color changes. (**A**) The color of a sample shifts from red to yellow during LAMP in positive samples. (**B**) Typical colors exhibited by positive samples throughout the LAMP reaction. (**C**) On-line determination of the change in color during LAMP. (**D**) Typical departure of color (from red to yellow) in negative samples (red trend), and positive samples containing 100 (yellow trend), 1000 (green trend), and 10000 (blue trend) copies of SARS-CoV-2 genetic material.

**Table S1.** Average electric potential associated with buffers of different pH values. The standard deviation and the coefficients of variance of the potential determinations, as determined during online determinations of 60 min (n = 1800), are also reported.

| pH value | Pot (mV) | STD | CV (%) |
|---|---|---|---|
| 6.00 | 2891.32 | 2.76 | 0.10 |
| 6.40 | 2843.71 | 6.91 | 0.24 |
| 6.80 | 2777.02 | 1.38 | 0.05 |
| 7.20 | 2713.54 | 1.38 | 0.05 |
| 7.60 | 2639.10 | 2.76 | 0.10 |
| 8.00 | 2587.58 | 6.21 | 0.24 |

**File S1: Arduino cod needed for LF-qLAMP**

**Arduino code**
(version1: considers the use of a of a Dallas-thermocouple)

//Arduino IDE code for the simultaneous control of temperature in a setpoint and determination of//electrical potential in the reaction mix during LAMP or PCR amplification.

```
//This version considers the use of a Dallas-thermocouple
//This program was assembled by Mario Moisés Alvarez at Tecnológico de Monterrey, México.
//To this aim, sections of code from various open sources were used:

//OPEN USE; PLEASE FOLLOW THESE RECOMMENDATIONS TO PROPERLY AKNOWLEDGE OUR LAB
//This code is intended for open use. If used, please cite the corresponding article if used.
//Please also acknowledge the Alvarez-Trujillo Lab, at Tecnológico de Monterrey, México.
//http://www.alvareztrujillolab.com/

//To run this code, several Arduino libraries must be downloaded and made accessible:
//--- <DallasTemperature.h> --- (for sinc with thermocouple)//https://github.com/collin80/dallas_temperature_control/blob/master/DallasTemperature.h
//--- <OneWire.h> ---    (for communication) https://github.com/PaulStoffregen/OneWire
//--- <PID_v1.h> ---   (for PID control) https://github.com/br3ttb/Arduino-PID-Library
//****************************************************************
//Arduino PID Library - Version 1.2.1
//by Brett Beauregard <br3ttb@gmail.com> brettbeauregard.com
//
//This Library is licensed under the MIT License
//****************************************************************
//We acknowledge the contribution of the authors of these libraries that enable this code

#include <PID_v1.h>
#include <Arduino.h>
#include <OneWire.h>
#include <DallasTemperature.h>

OneWire ourWire(4);                    //Se establece el pin 4   como bus OneWire
DallasTemperature sensors(&ourWire);//Se declara una variable u objeto para nuestro sensor

const int adcPin = A0;
const int sampleRate = 1;
double Setpoint, Input, Output;
#define   Kp 22.2
#define   Ki 0.00
#define   Kd 0.00

PID ControlPID(&Input, &Output, &Setpoint, Kp, Ki, Kd, DIRECT);

//Variables
float temperature_read = 0.0;
float time = 0;
float PID_error = 0;
float Setpointzero = 0;
float phVoltage1; float phVoltage2; float phVoltage3; float phVoltage4; float phVoltage5;

void setup() {

  pinMode(10,OUTPUT);

ControlPID.SetMode(AUTOMATIC);
ControlPID.SetSampleTime(sampleRate);
```

```
//Initialize SetPoint
//Setpoint to 62.5 degrees
   Setpoint = 62.5;
   Setpointzero= Setpoint;
//The value "6.5" is a correction factor to artificially increase the set point and compensate for heat
//losses in the incubator. Please modify accordingly to achieve your aimed set point.
   Setpoint= Setpoint + 6.5;
   Input=0;
   Output=0;
   phVoltage1 = 0.0;
   phVoltage2 = 0.0;
   phVoltage3 = 0.0;
   phVoltage4 = 0.0;
   phVoltage5 = 0.0;
   Serial.begin(9600);
}

void loop() {
float average;
   int adcValue = analogRead(adcPin);
   float phVoltage = (float)adcValue * 5000/1024 ;
   phVoltage5= phVoltage4;
   phVoltage4= phVoltage3;
   phVoltage3= phVoltage2;
   phVoltage2= phVoltage1;
   phVoltage1= phVoltage;
   phVoltage= (phVoltage1+phVoltage2+phVoltage3+phVoltage4+phVoltage5)/5;
uint8_t i;
average = 0.0;
int val=0;
  val = round(Output);
  analogWrite(10,val);

  sensors.requestTemperatures();    //Se envía el comando para leer la temperatura
          float temp= sensors.getTempCByIndex(0);//Se obtiene la temperatura en ºC
//+1.5 is a correction factor that should be modified as needed to conciliate Tread with Treactor
       temperature_read = temp +2.0;
//The value "2.0" is a correction factor that corrects for the difference in the temperature reading of//the thermocouple
in the bulk of the liquid in the incubator and the actual temperature of the reaction//mix in the Eppendorf tube.

//Next we calculate the error between the setpoint and the real value
PID_error = Setpointzero - temperature_read;
Input= temperature_read;
ControlPID.Compute();
   Serial.print("T :");
   Serial.print(temperature_read,1);
   Serial.print(" C ;    Po    = "); Serial.println(phVoltage, 3);
delay (942);
}
```

**Arduino code**
(version2: considers the use of a MAX6675 module for T sensing)

```
//Arduino IDE code for the simultaneous control of temperature in a setpoint and determination of//electrical potential in the reaction mix during LAMP or PCR amplification.
//This version of code considers the use of a MAX6675 module for T sensing
//This program was assembled by Mario Moisés Alvarez at Tecnológico de Monterrey, México.
//To this aim, sections of code from various open sources were used:

//OPEN USE; PLEASE FOLLOW THESE RECOMMENDATIONS TO PROPERLY AKNOWLEDGE OUR LAB
//This code is intended for open use. If used, please cite the corresponding article if used.
//Please also acknowledge the Alvarez-Trujillo Lab, at Tecnológico de Monterrey, México.
//http://www.alvareztrujillolab.com/

//To run this code, several Arduino libraries must be downloaded and made accessible:
//--- <SPI.h> ---      (for sinc with thermocouple) https://www.arduino.cc/en/reference/SPI
//--- <Wire.h> ---      (for communication) https://www.arduino.cc/en/reference/wire
//--- <PID_v1.h> ---    (for PID control) https://github.com/br3ttb/Arduino-PID-Library
//***************************************************************
//Arduino PID Library - Version 1.2.1
//by Brett Beauregard <br3ttb@gmail.com> brettbeauregard.com
//
//This Library is licensed under the MIT License
//***************************************************************
//We acknowledge to the authors of these libraries that enable this code

//    This embodiment of the program uses a MAX6675 module for temperature reading
// Max6675 Module    ==>      Arduino
//    CS                    ==>         D6
//    SO                    ==>         D12
//     SCK                    ==>        D7
//     Vcc                   ==>        Vcc (5v)
//     Gnd                    ==>        Gnd

#include <Wire.h>
#include <PID_v1.h>
#include <Arduino.h>
const int adcPin = A0;
const int sampleRate = 1;
double Setpoint, Input, Output;

#define    Kp 22.2
#define    Ki 0.00
#define    Kd 0.00

PID ControlPID(&Input, &Output, &Setpoint, Kp, Ki, Kd, DIRECT);

#include <SPI.h>
//We define the SPI pìns
#define MAX6675_CS      6
```

```
#define MAX6675_SO      12
#define MAX6675_SCK    7
#define NUMSAMPLES 5


//Variables
float temperature_read = 0.0;
float time = 0;
float PID_error = 0;
unsigned long now;
float Setpointzero = 0;
float phVoltage1; float phVoltage2; float phVoltage3; float phVoltage4; float phVoltage5;
int counter1 = 0;

void setup() {
  pinMode(10,OUTPUT);
ControlPID.SetMode(AUTOMATIC);
ControlPID.SetSampleTime(sampleRate);
//Initialize SetPoint
//Setpoint to 62.5 degrees
   Setpoint = 62.5;
   Setpointzero= Setpoint;
//The value "6.5" is a correction factor to artificially increase the set point and compensate for heat
//losses in the incubator. Please modify accordingly to achieve your aimed set point.
   Setpoint= Setpoint + 6.5;

   Input=0;
   Output=0;
   phVoltage1 = 0.0;
   phVoltage2 = 0.0;
   phVoltage3 = 0.0;
   phVoltage4 = 0.0;
   phVoltage5 = 0.0;
   Serial.begin(9600);
}

void loop() {
float average;

   int adcValue = analogRead(adcPin);
   float phVoltage = (float)adcValue * 5000/1024 ;
   phVoltage5= phVoltage4;
   phVoltage4= phVoltage3;
   phVoltage3= phVoltage2;
   phVoltage2= phVoltage1;
   phVoltage1= phVoltage;
   phVoltage= (phVoltage1+phVoltage2+phVoltage3+phVoltage4+phVoltage5)/5;
// Serial.println (phVoltage5);
// Serial.println (phVoltage4);
// Serial.println (phVoltage3);
// Serial.println (phVoltage2);
// Serial.println (phVoltage1);
```

```
uint8_t i;
average = 0.0;
int val=0;
//First we read the real value of temperature
//take N samples in a row, with a slight delay
      for (i=0; i< NUMSAMPLES; i++) {
        samples[i] = readThermocouple();
//        Serial.println(readThermocouple());

        delay(10);
      average/= NUMSAMPLES;//calculate average
}
  val = round(Output);
  analogWrite(10,val);

temperature_read = readThermocouple()+2.0;
//The value “2.0” is a correction factor that corrects for the difference in the temperature reading of//the thermocouple in the bulk of the liquid in the incubator and the actual temperature of the reaction//mix in the Eppendorf tube.

//Next we calculate the error between the setpoint and the real value
PID_error = Setpointzero - temperature_read;
//Serial.println(PID_error,1);

Input= temperature_read;

      ControlPID.Compute();
// Serial.println(val);

    Serial.print("T :");
    Serial.print(temperature_read,1);
    Serial.print(" C ;    Po    = "); Serial.println(phVoltage, 3);
    counter1 = counter1 + 1;
    if (counter1 == 5) mV_baseline = phVoltage;
    if (counter1 > 5) {
       deltamV = phVoltage - mV_baseline;
    }
delay (942);
}



double readThermocouple() {

    uint16_t v;
    pinMode(MAX6675_CS, OUTPUT);
    pinMode(MAX6675_SO, INPUT);
    pinMode(MAX6675_SCK, OUTPUT);

    digitalWrite(MAX6675_CS, LOW);
    delay(1);
```

```
//Read in 16 bits,
// 15      = 0 always
// 14..2 = 0.25 degree counts MSB First
// 2        = 1 if thermocouple is open circuit
// 1..0   = uninteresting status

  v = shiftIn(MAX6675_SO, MAX6675_SCK, MSBFIRST);
  v <<= 8;
  v |= shiftIn(MAX6675_SO, MAX6675_SCK, MSBFIRST);

  digitalWrite(MAX6675_CS, HIGH);
  if (v & 0 × 4)
  {
   //Bit 2 indicates if the thermocouple is disconnected
     return NAN;
  }

//The lower three bits (0,1,2) are discarded status bits
  v >>= 3;

//The remaining bits are the number of 0.25 degree (C) counts
  return v*0.25;
}
```
